# Public Attitudes, Preferences and Willingness to Pay for River Ecosystem Services

**DOI:** 10.3390/ijerph16193707

**Published:** 2019-10-01

**Authors:** Imran Khan, Hongdou Lei, Gaffar Ali, Shahid Ali, Minjuan Zhao

**Affiliations:** 1 College of Economics and Management, Northwest A&F University, 3 Taicheng Road, Yangling 712100, China; leihongdou@nwafu.edu.cn; 2 Department of Agricultural & Applied Economics, The University of Agriculture, Peshawar 25130, Pakistan; ghaffarali@aup.edu.pk (G.A.); drshahid@aup.edu.pk (S.A.)

**Keywords:** Ecosystem services, Environmental awareness, Environmental policy, Public attitude, Random parameter logit (RPL) model, Water quality

## Abstract

River basins are key sources of ecosystem services, with a wide range of social and economic benefits and many effects on human well-being. However, intensified land use and other dramatic variations in river ecosystems can alter ecosystem functions and services. In this study, we explored the public awareness, attitude and perception regarding environmental and water resource issues and assessed the willingness to pay (WTP) for improving selected attributes of the Wei River basin. Various rankings, Likert scales and random parameter logit (RPL) models were used to analyze data obtained from 900 surveyed respondents. Most respondents were more concerned about environmental and water resource management issues rather than socioeconomic attributes. From a policy perspective, 83.32% and 50.50% of the residents ranked “improvement in water quality” and “improving irrigation conditions,” respectively, as their main priorities regarding ecological restoration. Moreover, the results obtained using RPL models showed that the coefficients were significant for all ecological attributes and monetary attributes, as expected. The positive and significant coefficient for the alternative specific constant demonstrated that the respondents preferred restoration alternatives to the status quo. Furthermore, the highest WTP was found for water quality (91.99 RMB), followed by erosion intensity (23.59 RMB) and water quantity (11.79 RMB). Our results are relevant to policy development and they indicate that ecological restoration is the favored option.

## 1. Introduction

From a social perspective, river basins are major sources of ecosystem services, with a wide range of economic benefits and they have diverse effects on human well-being [1,2]. Ecosystem services are the direct or indirect benefits provided by biodiversity and ecosystems to humans [3,4]. The concept of ecosystem services has gradually gained prominence in the context of sustainable natural resource management [1,5,6,7,8]. Ecosystems provide cultural, regulatory and support services that directly or indirectly promote human well-being through recreation, landscape values and fisheries maintenance [9,10,11]. However, the intensification of land use as well as channelization, damming and other drastic changes to river ecosystems can alter the functions and associated services provided by river ecosystems [12,13,14]. Understanding the social and economic value of river ecosystem services can help to shape the priorities for river restoration projects [15,16,17,18]. Consequently, it is essential to enhance public awareness regarding the importance of river ecosystem services and mitigate current trends [19,20].

In addition to identifying, classifying and conducting ecological assessments of the functions and services provided by river ecosystems, the sustainable management of water resources can help to utilize the non-market-effective monetary valuations associated with river ecosystem services [21,22]. However, public organizations rarely treat river restoration projects as investments at present [23]. The funds available to restore natural capital are far lower than those for building and maintaining building infrastructure. Reframing river restoration projects as the recovery of natural capital may attract the financial resources needed to reestablish degraded river ecosystems [24,25] and better integrate social and environmental values [26]. In order to promote socially optimal decision making regarding river ecosystem restoration, non-market benefits can be determined based on the economical use of these ecosystems or their services [27,28]. The validity of non-market assessment techniques is based on the type of benefit estimates employed and the use or non-use value of the participating valuation background [29,30].

Over recent decades, the emphasis on the value of ecosystem services has increased [31,32,33] because this value may influence effective public environmental policies [34]. The monetary value of water resources provides all the necessary information [35,36], including water availability, water quality and its alternative uses, to help decision-makers [37,38] obtain cost and benefit estimates for any developmental project [39]. Moreover, the economic valuation of ecosystem services helps to link human behavior and policy making regarding natural ecosystems [40,41], although few studies have investigated this link [42,43]. Many studies have aimed to explain the importance and values of natural areas for humans (e.g., References [1,32,44,45,46]). Numerous studies have emphasized individual environmental values and others have considered the willingness to pay (WTP) for environmental protection [1,47].

Several methods can be used to conduct monetary assessments of ecosystem services and to elicit the public’s WTP for ecosystem restoration [4,48,49]. WTP is a common concept employed in numerous approaches for determining the amount of money that individuals have spent or are hypothetically willing to pay to use, improve or restore ecosystem services or natural resources [33,50]. According to previous studies by Khan, et al. [4] and Nicosia, et al. [50], the WTP may be calculated by considering the revealed preferences or stated preferences approach, where the former evaluates the amount of money paid by societies for using a specific resource [51], whereas the latter elicits the WTP directly or indirectly by using surveys [48]. The contingent valuation and choice experiment approaches both consider hypothetical market scenarios and they employ surveys to determine user preferences [4,52].

In the present study, we used a spatially explicit choice experiment technique to evaluate the monetary values of ecosystem services by asking respondents how much they would be willing to pay for improving or restoring degraded ecosystem services [4,50,53]. This technique also permits the assessment of non-use values [54]. From the perspective of Chinese society, the potential benefits of conservation enhancements in the Wei River Basin are generally unexplored [55]. Thus, assessing the potential welfare estimates obtained from improving river ecosystem services is essential [56,57,58]. If we do not value these services, then the fiscal system upon which we depend will continue to favor the over-exploitation of resources and degradation of ecosystem services [55,59]. Estimates of possible benefits can be employed in cost-benefit analyses for governance projects in the Wei river basin.

In particular, in this study, we aimed to determine the links between how people value water resources and their attitudes, preferences for water resources and their WTP to restore or improve highly degraded river basin ecosystems based on an approach for evaluating changes in the quality and quantity of inland river resources. Moreover, our results can be used in benefit transfer analysis to determine the social welfare benefits obtained from improving river ecosystems in similar river basins [4,55,60].

The specific objectives of this study were as follows. First, we aimed to determine the importance of ecological and water resource amenities and to establish a model for determining the range of monetary values provided by different groups of people for river ecosystems. Second, we attempted to determine the relative importance of various ecological issues to different groups of people living in a specific area and to identify the key factors that might affect the WTP for river ecosystems. Finally, we determined the environmental and socioeconomic characteristics that might affect individual preferences regarding the ecological protection and future management of rivers.

## 2. Materials and Methods

### 2.1. Study Area

**Figure 1:**
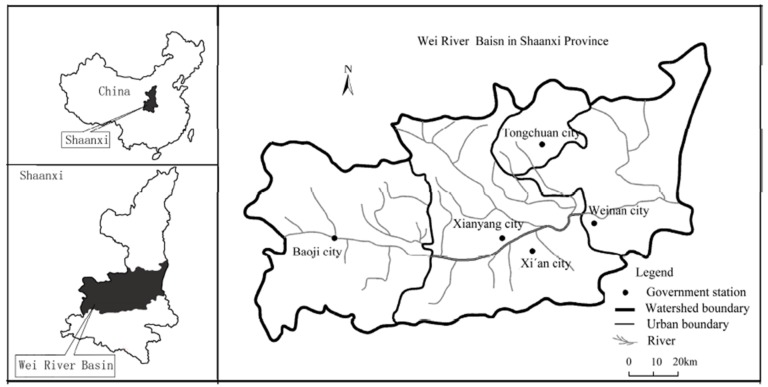
Map of the study area.

The largest and highly sandy tributary of the Yellow River called the Wei River is located in the arid and semiarid region of Northwest China [61], as shown in Figure 1, where it flows through three geographical areas comprising the Loess plateau—Guangzhong basin in Shaanxi province and the North Qinling Mountains [62]. The Guangzhong area is located in the middle and lower reaches of the Wei River and it covers almost 135,000 km^2^ (about 50%) of the entire river basin [55,63]. Due to its fertile land and well-developed irrigation system, the Guangzhong area plays an important role in food production in China. The Wei River still plays a significant role in the development of Western China in general and Shaanxi province in particular [64,65]. However, due to rapid economic growth and rapid urbanization during the last few decades, environmental issues are increasing because of the growing utilization of water resources [55]. According to the Wei River Key Governance Plan, the main ecological problems include the scarcity of water resources [66], land use and climate change [67], soil erosion and water loss [55], regional ecological environment degradation and water pollution [62]. Due to severe environmental problem and water scarcity, the average per capita water consumption is only 13% of the national average. The Wei River basin is highly degraded because of the increasing amounts of wastewater discharged mainly from major cities in Shaanxi, such as Baoji, Xian yang, Xian and Weinan. Furthermore, large amounts of industrial wastewater flow directly into the river without processing because effective pollution treatment systems have not been installed by local industries. The Wei River basin was selected as the focus of the current study because of these problems related to river degradation.

### 2.2. Choice Experiment

Studies have shown that, compared with other valuation techniques, choice experiments provide more robust estimates of the value of non-market goods and services [68], particularly when estimating improvements in environmental quality. According to Bateman, et al. [69], the choice experiment technique uses a survey instrument to represent environmental goods and services as a set of attributes, where the levels of the attributes vary across the choice set, thereby allowing assessments of how individuals react to different attribute levels [70]. Many studies, such as those by Morrison, et al. [71] and Carlsson, et al. [72], have applied the choice experiment approach for evaluating non-market goods and services in wetlands and their findings demonstrated that the choice experiment approach can improve the understanding of ecological attributes, help policymakers by providing information about the values of various options and identify the ecological attributes that influence the perceived values of wetlands for individuals. Choice experiments apply random utility theory to determine whether an individual chooses a conservation program or wetland ecosystem among J alternatives [6,70]. The associated utility that an individual obtains from choosing alternative j among J alternatives is U_nj_, j= 1...J and it is assumed that a rational individual will only choose an alternative that gives him/her the maximum possible utility [4]. According to Train [73], an individual i will prefer and choose an alternative j over k if and only if the associated utility of choosing alternative j is greater than alternative k, that is, U_ij_ > U_ik_. The utility for an individual i from choosing alternative j has two components, that is, a deterministic component V_ij_ and stochastic component ε_ij_, as follows:

(1) $$U_{ij}=V_{ij}+\varepsilon_{ij}$$

where U_ij_ is the latent utility or unobservable utility of the chosen alternative, V_ij_ is the systematic observable component or deterministic component of utility and ε_ij_ is the unobservable or stochastic component of latent utility with alternative j and individual i.

The joint density of a random vector, ε_i_, leads to an expression for the probability of choice [73]. Mathematically, it is computed as follows:

(2) $$P_{ij}=\mathrm{Pr}ob\left[\left(V_{ij}+\varepsilon_{ij})>(V_{ik}+\varepsilon_{ik}\right)\right]$$

OR

(3) $$P_{ij}=\mathrm{Pr}ob\left[\left(\varepsilon_{ik}-\varepsilon_{ij})<(V_{ij}-V_{ik}\right)\right]$$

OR

(4) $$P_{ij}=\int_{\varepsilon }I(\varepsilon_{ik}-\varepsilon_{ij}<V_{ij}-V_{ik})f(\varepsilon_{i})d\varepsilon_{i}$$

for all k ≠ j, where P_ij_ denotes the individual’s probability of choosing an alternative j. The indication function I(.) is equal to 1 if the expression in parenthesis is true and 0 otherwise. This is a multidimensional integral over the density of the unobserved portion of utility, f (ε_i_).

To account for potential correlation errors, we employed the mixed logit (random parameter logit (RPL) model), which assumes that the coefficients (β_n_) of variables are random. The RPL model offers full relaxation of the independence of irrelevant alternatives (IIA) in the multinomial logit model [74] and it allows the parameters to vary randomly among the respondents [75,76]. The RPL also assumes that unobserved factors affect the utility. The mixed logit model is more flexible than conditional logit and multinomial logit models [70,77,78,79] and it allows for heterogeneous preference substitutions and correlations among unobserved factors [4,55]. The utility obtained by an individual i from choosing an alternative j is quantified as:

(5) $$V_{ij}=ASC+\beta_{i}x_{ij}+\varepsilon_{ij}$$

where *ASC* represents the alternative specific constant, X_ij_ denotes the observed variables, β_i_ denotes the vector of the coefficients for these variables for an individual i representing his/her preference heterogeneity, which varies among people rather than being fixed [73] and ε_ij_ is a random term. The unconditional probability is the integral of Equation (6) over all possible variables of β_i_.

(6) $$P_{ij}=\int \left[\frac{e^{\beta^{\prime }x_{ij}}}{\sum_{k}e^{\beta^{\prime }x_{ik}}}\right]f(\beta )\partial \beta$$

As suggested by Train [73] and others (e.g., Morey, et al. [79] and Carlsson, et al. [72]), f(β)was assumed to be normally distributed in our model. The RPL model considers the correlations of error terms when the coefficients β_s_ are allowed to be normal. Hence, we assumed that unobservable elements can affect the choices of other attributes (e.g., improving the water quantity and quality will also increase vegetation cover and wildlife habitat) and thus the preferences of the individuals. Considering the heterogeneity of preferences in a model will lead to unbiased estimates and help to enhance the reliability and accuracy of the welfare estimates [80,81]. Previous studies have demonstrated that the RPL model is superior to the conditional logit and multinomial logit models in terms of welfare estimates and the overall fit [4,70,77,79,82].

The WTP estimates obtained for any improvements in ecological attributes using the choice experiment approach are based on the implicit prices for alternative ecosystem service choices given that others factors remain constant [83]. The marginal rate of substitution between the preferred ecological attribute and a monitory attribute determines the implicit price of that specific ecological attribute [84]. The implicit price is computed based on the ratio of the coefficient for the ecological attribute (one of the non-monitory attributes) β_k_ relative to the coefficient for the monitory attribute, that is, β_p_, as follows:

(7) $$Implicit\_price=-(\beta_{Non-monetary\_attribute}/\beta_{monetary\_attribute})$$

OR

(8) $$WTP=-(\beta_{k}/\beta_{p})$$

The ecological attributes of the Wei River were identified based on previous studies and discussions with other researchers and local representatives. The main ecological attributes of the Wei River and their levels, which we aimed to estimate, are shown in Table 1, that is, the forest cover ratio, water quality, water quantity, erosion area (the amount of controlled soil and water loss area), erosion intensity, natural landscape and conditions for eco-tourism and parks.

### 2.3. Data Collection

A survey was conducted to collect the data for the present study, where pre-tested and well-designed questionnaires were used to reach the entire samples of major cities and counties throughout the study area. Two trial surveys were conducted before designing the final questionnaire in order to examine the cognitive complexity of the respondents and by considering the trade-offs in the differences among the indicators. The survey was performed by well-trained university graduates. Face-to-face interviews were held due to the complexity of the questionnaire and in order to include rural and less well-educated people. The interviewers could assist the respondents by providing clarifications, thereby reducing the likelihood of problems associated with understanding the questionnaire. The introductory part of the questionnaire identified a respondent’s connection with the sites, their perception of the importance of ecosystem services and their attitude toward the improvement policy for ecological attributes. The choice experiment in the Wei River was designed to obtain practical welfare estimations. In order to ensure that accurate parameters were estimated, the D-optimal orthogonal design in SAS software was employed to develop the choice alternatives. Based on one status quo and two policy scenarios, a total of 512 choice sets were generated. After testing for unreliable and dominant choice sets, 450 choice sets were clustered into 150 booklets with three choice tasks in each. Each respondent was asked to select his/her preferred alternative from a total of three independent choice tasks. The subjects were told that the payment associated with the policy choice was a mandatory annual payment for a 10-year period, which would be applied as special funds and only used for ecological improvement in the basin.

In the current study, a multistage sampling technique was employed to collect the primary data from participants throughout the entire basin. In the first sampling stage, the Shaanxi section of the basin was selected due to its agricultural and ecological importance and three integral parts were identified based on the pre-existing administrative divisions, that is, upper, middle and lower sub-basins. In the second stage, the main cities (prefecture administrative level) in these sub-basins were selected as the references points. Based on these reference points, the townships followed by random sample villages were randomly selected and the sample respondents were drawn randomly. In the third stage, the participants were categorized into two groups comprising urban and rural households. Sample representatives were selected from each group using a stratified random sampling technique. Urban and rural households were selected randomly from residential areas and villages, respectively. Finally, 12 to 15 respondents were selected randomly from these communities, thereby resulting in a total of 900 households, as shown in Table 2.

## 3. Results and Discussion

### 3.1. Rankings of Importance for Socio-Economic and Environmental Issues

**Figure 2:**
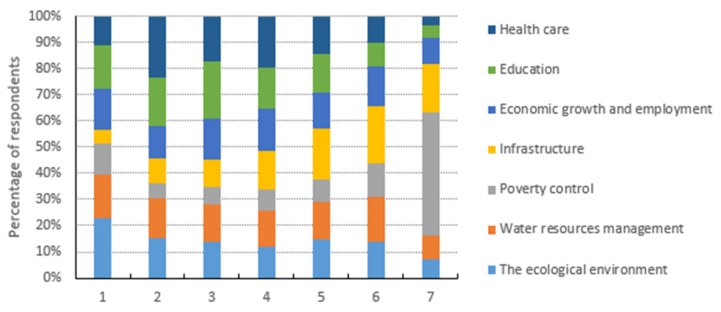
Rankings of importance for socio-economic and environmental issues.

The subjects were asked to rank the socioeconomic and ecological issues based on their relative importance and preferences, such as the ecological environment, water resource management, infrastructure, economic growth and employment, education and health care, where they assigned scores ranging from 1 denoting “most important” to 7 representing “least important.” As shown in Figure 2, the majority of the inhabitants of the Wei river basin were more concerned about the ecological environment and water resource management rather than the socioeconomic attributes. We found that 23% of the respondents considered the ecological environment as their main priority, followed by 17% of the respondents who valued water resource management as their key priority. Similarly, 38% of the total respondents (23% considered as first and 15% as second) valued the ecological environment as their highest and second highest priority, followed by 32% (17% and 15%) who considered water resource management as their highest and second highest priorities. In addition to ranking the ecological environment and water resource management as the highest and second highest preferred attributes, the inhabitants of the Wei River basin perceived and ranked poverty reduction and infrastructure as their least important issues. The detailed rankings of all the socio-economic and ecological attributes are shown in Figure 2.

Table 3 shows the mean values, standard errors, 95% confidence intervals and side by side ranking comparisons for the socio-economic and environmental issues. The residents of the Wei River basin rated education, health care, ecological environment and water resource management as their four most important issues with mean values of 3.39, 3.46, 3.51 and 3.81, respectively. The results also indicated that poverty reduction and infrastructure had mean values of 5.22 and 4.74, respectively and they were considered the least important issues. Due to considerable reductions in poverty [62,85] and the construction of infrastructure [86,87] in the last 45 years, poverty is no longer an issue in Northwest China but this achievement has been obtained at the cost of environmental degradation [64,65] and health risks [88].

### 3.2. Public Concern about Ecological and Water Resource Issues

**Figure 3:**
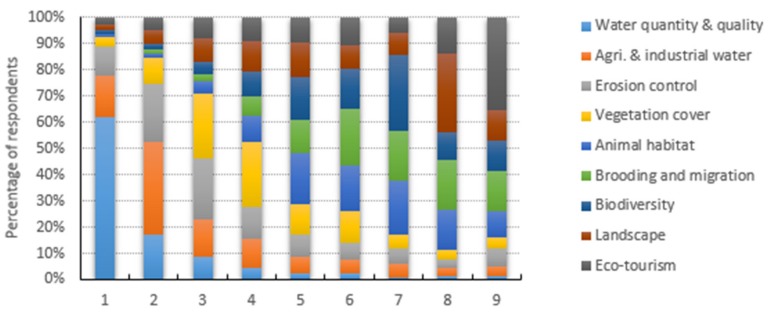
Rank score of Wei river respondents for ecological attributes from most important “1” to least important “9.”.

In Northwest China, the Wei river basin is regarded as a hub of related ecological and water resource issues. In addition to the choice options, our questionnaire included a ranking question regarding the prevailing ecological and water-related issues faced by residents throughout the entire basin. The issues identified were based on extensive literature review and discussions with university researchers, relevant scientists, the pilot group and administrative bodies in the Wei River basin. The surveyed respondents were asked to rank these ecological and water-related issues from most concerning with “1” to least concerning with “9” based on their perceptions. Thus, Figure 3 shows the rank scores for ecological attributes based on their relative importance to residents in the Wei River basin. The results showed that 61.9% of the respondents considered water quantity and quality as their key concerns and 35.6% considered agricultural and industrial water as their second most concerning ecological issue in the Wei River basin. In addition, erosion control (soil and water loss), vegetation cover and biodiversity were highly concerning ecological attributes. Moreover, the results demonstrated that ecotourism, followed by brooding and migration, animal habitat and landscape deterioration were the least concerning ecological issues (Figure 3).

Table 4 shows the mean values, standard errors, 95% confidence intervals and comparisons of the rank scores for ecological attributes based on the concerns of the respondents. As shown in Table 4, the residents of Wei river basin were most concerned about ecological issues where they preferred improvements in water quantity and quality, agricultural and industrial water use, erosion (soil and water loss) and vegetation restoration with mean values of 1.93, 3.30, 3.85 and 4.31, respectively. The results also indicated that the respondents were less concerned about ecological issues with a mean value above 6. For example, the residents of the Wei River basin were less concerned about ecological attributes such as brooding and migration, ecotourism, biodiversity, animal habitat and landscape deterioration with mean values of 6.61, 6.54, 6.19, 6.16 and 6.08, respectively.

### 3.3. Concerns about Policy Implications of River Ecological Attributes

**Figure 4:**
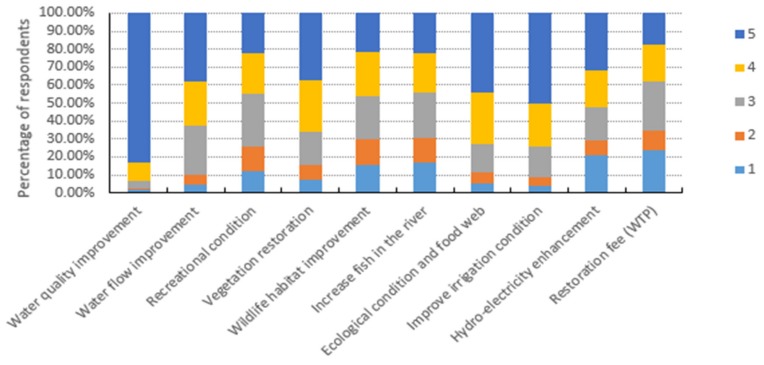
Importance of environmental policy interventions to improve ecological amenities, where the perception rankings ranged from “5” for very important to “1” for not important.

In our survey questionnaire, the respondents in the Wei River basin were asked about the importance and their concerns regarding policy interventions to improve the ecological attributes of the significantly degraded Wei River basin. The surveyed respondents were asked to rank the importance of improvements in the ecological attributes of the Wei River (policy interventions) from 1 as not important to “5” as very important based on their perception and knowledge. Figure 4 shows the ranking preferences for policy interventions to improve the river ecosystem services. As shown in Figure 4, the majority of the respondents, that is, 83.32%, considered that improving water quality was a very important policy intervention and 9.85% of the respondents considered it an important policy, probably because the river water quality is highly degraded in most river basins in China [2,88].

Similarly, 50.50% of the residents considered that improving irrigation conditions was the most important intervention and 24% of the households considered it an important policy intervention. Furthermore, the results showed that ecological conditions and food web, water flow improvement, vegetation restoration and hydro-electricity enhancement were the next most important policy issues. However, the restoration fee was considered the least important or a not important policy issue (Figure 4).

Table 5 shows the means, standard errors and 95% confidence intervals for the rankings of importance given to various ecological restoration policies. As shown in Table 3, water quality, irrigation condition and ecological condition and food web were the most important policy issues with mean values of 4.73, 4.12 and 4.01 respectively. Similarly, except for the restoration fee, all of the ecological attributes had a mean value above 3 and thus they ranged from average importance to important policy issues.

### 3.4. Estimated Results Obtained Using the Mixed Logit Model

Table 6 shows the results obtained with the mixed logit model, which used a simulation likelihood with 500 Halton draws to specify the models to account for the correlations between the survey responses for each respondent. The choice experiment model was specified so the probability of selecting a specific alternative policy regarding river ecosystem restoration was a function of ASC and the river ecological attributes for that policy alternative. ASC was assumed to be 1 if the survey respondent selected a policy alternative (A or B) and 0 if the respondents selected the status quo alternative from among the given set of alternatives. Using 8100 choices elicited from 900 respondents, a mixed logit model was assessed using Stata Statistical Software, version 14, (College Station, TX: StataCorp LP). It should be noted that based on the Hausman–McFadden test and the large values for the chi-square test, the assumption of homoscedasticity was violated and the IIA restriction was rejected [89], which allowed the application of a more flexible mixed logit model. Our model assumed a normal distribution and all of the ecological attributes were specified as randomly distributed, except for payment and ASC, which we specified as fixed. The results in Table 6 show that all of the coefficients obtained from the RPL model were highly significant at the 1% level and the signs agreed with our expectations. As suggested by Train [73] and Hole [90], no constraint was applied on the signs of the ecological attributes with random parameters. The highly significant coefficient for the mean standard deviation indicated that the respondents had heterogeneous preferences [4,55]. All of the river ecological indicators were highly significance in the choice set of river restoration scenarios and a higher level for any single ecological indicator affected the probability of selecting that management scenario (alternative) when other factors remained the same (e.g., site attachment, preference heterogeneity, socioeconomic characteristics and substitute availability). The positive and statistically significant coefficient for ASC demonstrated that the respondents preferred the alternative states for restoration rather than status quo and any move away from the status quo obtained a positive utility impact [91,92]. Hence, in general, the outcomes showed that the respondents were willing to pay annually to move away from the “no action” (status quo) alternative if all other factors remained constant.

### 3.5. Estimation of WTP or Implicit Prices

Consistent with demand theory, the implicit prices or marginal WTP can be computed based on the estimated coefficients generated using RPL model. As described by Birol, et al. [78] and Shi, et al. [55], and following the method of welfare simulation [74], we calculated the marginal rate of substitution between the change in a particular ecological indicator and the marginal utility of the payment attribute (income) using Equation 8. As suggested by Khan, et al. [6], the Wald procedure (Delta method) was applied to determine the marginal WTP for each river ecological indicator and the results are shown in Table 7. The projected WTP values represent the amount that an individual would be willing to pay per year for one unit/level improvement in a specific ecological attribute while other factors remain constant. For example, the implicit price (WTP) for forest cover ratio was 9.87, which means that an individual was willing to pay RMB 9.87 per year for one unit improvement in the current level of forest cover. Similarly, the highest WTP, was found for water quality, that is, RMB 91.99, followed by erosion intensity (23.59) and water quantity (11.79), which indicate that the respondents were willing to pay RMB 91.99 for one unit improvement in the water quality (e.g., from level 4 to level 3) per year, followed by RMB 23.59 for one unit decrease in the erosion intensity and RMB 11.59 for a 1% increase in the current water quantity, respectively.

Our results confirmed that improving the water quality was the most desirable river ecosystem service for the sampled respondents. Society and policymakers are increasingly recognizing the roles of watersheds in providing clean water [93]. Hence, society should support public policies that encourage the enhancement of water bodies with the objective of preserving or improving the water quality. The negative average coefficients for water quality and erosion intensity are related to the units of measurement because lower values are preferred and the expected negative average cost factors infer the positive utility of money.

### 3.6. Policy Implications and Future Directions

Some key policy implications emerged based on our findings. In particular, the need to enhance the natural river ecosystems and ensure the security of water resources means that the Chinese government should strictly prevent further degradation of these estuaries and develop specific policies to improve the ecological attributes of rivers. Suitable water programs and strategies should be designed in order to meet the demand for water resources. Moreover, water is a public good and it should be made available to consumers in the best possible condition and thus users need to be educated about its significance in their lives to ensure sustainable water use. The calculated marginal utility that the inhabitants would derive from improvements in the ecological conditions in the Wei River basin varied considerably among households, possibly due to disparities in the economic and environmental attributes throughout the study area. Hence, the current spatial heterogeneity should be considered when formulating environmental protection policies. In order to achieve substantial improvements in the environment and water resources, the Chinese Government launched the Water Pollution Control Action Plan, which emphasizes the need for public participation in water and ecological protection. However, this plan fails to consider the heterogeneity in terms of people’s preferences regarding improvements to river ecosystems. Thus, the results obtained in the present study should provide policy makers with valuable information to facilitate the management of ecosystem services. Our findings provide guidance, policy recommendations and references for researchers in order to improve and enhance the current river water services in the future. Further studies are needed to confirm our results and to investigate the factors that influence the heterogeneous preferences of people who live in various river basins.

### 3.7. Limitations of the Study

Although the present study demonstrates potential contributions to the existing literature, it has several limitations. For example, using money as a mode of contribution may not capture the potential contribution of low-income households and hence further research is required with the option of contributing towards the ecosystems restoration in labor terms if they are unwilling to participate in monetary terms. All empirical results must be viewed within the context of the present case study and sample, along with the implied assumptions and possible limitations, for example, the results derived from a choice experiment are site-specific and focusing on a limited geographical area. Given the spatial heterogeneity of preferences for river ecosystem services and heterogeneity in China’s population (e.g., urban vs. rural), any conclusions regarding the general applicability of our findings will require additional work to validate these results elsewhere. Moreover, a comprehensive cost-benefit analysis is indispensable before definitive conclusions can be drawn regarding the efficiency of any particular policy, including those for river ecosystems improvements. One of the main limitations is the fact that for practical reasons and despite the operationalization of the framework of physical-material and symbolic-emotional dimensions of societal relations to nature, no other concepts like ecosystem services were used. Finally, although the present study includes a wide array of best practices for Stated Preferences design, there is always the possibility of additional improvements to enhance validity and reliability. Although illustrated for a particular region in northeast China, similar results could be applied elsewhere to provide a more comprehensive perspective on the public’s WTP for improving the degraded river ecosystems.

## 4. Conclusions

River basins are major sources of ecosystem services, which provide a wide range of social and economic benefits, with various effects on human well-being. However, rapid economic growth, vast infrastructure development, significant reductions in poverty, population growth and the increasing urbanization of the Chinese transitional economy have caused the continuous degradation of ecological and water resources, thereby affecting river ecosystem functions and their associated ecosystem services. Ecological degradation is one of the most pressing challenges in China and it threatens to undermine the country’s economic growth. At present, policymakers need to take actions to prevent further degradation, which requires more quantitative information to ensure that appropriate actions are taken to mitigate ecosystem-related problems. In this study, we used several rankings, Likert scales and RPL models to investigate the public awareness, attitudes and perceptions regarding river ecology and water resource issues, as well as assessing the WTP for ecological restoration. We found that the subjects were more concerned about the river ecological environment and water resource management rather than socioeconomic attributes. For example, 23% of the respondents stated that the ecological environment was their main priority, whereas 17% of the respondents stated that water resource management was their key priority. Similarly, 38% and 32% of the respondents ranked the “ecological environment” and “water resource management,” respectively, as their highest and second highest priorities. In addition, most of the respondents ranked “poverty reduction” and “infrastructure” as their least important issues.

Based on the relative importance of the ecological attributes, the ranking scores showed that 61.9% of the respondents considered water quantity and quality as most important and 35.6% considered agricultural and industrial water as the second most important ecological attribute. In addition, erosion control, vegetation cover and biodiversity were ranked highly, whereas ecotourism, brooding and migration, animal habitat and landscape deterioration were the lowest ranked ecological issues. From the perspective of ecological restoration, 83.32% and 50.50% of the respondents ranked “improvement in water quality” and “improving irrigation conditions,” respectively, as the most important policy implications.

The results obtained using the RPL model demonstrated that the coefficients for all of the attributes were highly statistically significant and their signs were as expected. The preferences of the respondents regarding improvements in various ecological attributes derived from the survey data and their behavior choices were analyzed to infer the WTP. In particular, among the ecological attributes included in the choice experiment, the respondents assigned the greatest value to water quality, that is, the highest WTP was found for water quality (91.99 RMB), followed by erosion intensity (23.59 RMB) and water quantity (11.79 RMB) and thus the respondents were willing to pay 91.99 RMB for one unit improvement in the water quality per year, 23.59 RMB for one unit decrease in the erosion intensity and 11.59 RMB for a 1% increase in the current water quantity. From a social perspective, these results are relevant to policymakers and they indicate that ecological restoration is the most important concern.

## Figures and Tables

**Table 1:** Attributes and levels in Wei River basin.

Attributes	Levels
Forest cover ratio	30%; 31%; 33%; 35%
Level of water quality	4.5; 4; 3.5; 3
Amount of water per capita (proportions of the national average)	15%; 17%; 19%; 20%
Amount of controlled soil and water loss area	80%; 85%; 88%; 90%
Erosion intensity	moderate (= 3); mild (= 2); light (= 1)
Natural landscape	20%; 25%;30%; 35%
Conditions for eco-tourism and parks	25%; 30%; 35%
Costs per household each year	0; 50; 100; 150; 200; 250; 300

**Table 2:** Sample distribution in Wei River basin.

Study Area	Citizen	Farmers	Total
Baoji	115	115	230
Xian yang	95	125	220
Weinan	102	121	223
Huayin	114	113	227
Total	426	474	900

**Table 3:** Mean and standard errors of rankings for socio-economic and environmental issues.

Socio-Economic and Environmental Issues	Mean	Standard Error	95% Confidence Interval	
Ecological environment for the resident	3.5077	0.0661	3.3780	3.6375
Water resource management	3.8077	0.0653	3.6796	3.9359
Poverty reduction	5.2177	0.0722	5.0762	5.3594
Infrastructure (highway, service facilities, etc.)	4.7366	0.0596	4.6198	4.8536
Economic growth and employment	3.8611	0.0642	3.7352	3.9870
Education	3.3922	0.0574	3.2796	3.5048
Health care	3.4656	0.0550	3.3575	3.5735

**Table 4:** Mean and standard errors for the rankings of ecological attributes.

Ranking Scores for Ecological Attributes	Mean	Standard Error	95% Confidence Interval	
Water quantity and quality	1.9333	0.0555	1.8243	2.0423
Agricultural and industrial water	3.2989	0.0718	3.1578	3.4399
Soil and water loss (erosion) control	3.8489	0.0764	3.6990	3.9988
Vegetation restoration	4.3133	0.0630	4.1896	4.4370
Animal habitat	6.1566	0.0595	6.0399	6.2734
Brooding and migration	6.6111	0.0577	6.4979	6.7243
Biodiversity	6.1989	0.0605	6.0801	6.3177
Landscape	6.0800	0.0755	5.9318	6.2282
Eco-tourism	6.5400	0.0825	6.3780	6.7020

**Table 5:** Ranking of importance for various ecological restoration policies.

Ecological Degradation /Environmental Issues	Mean	Standard Error	95% Confidence Interval	
Water quality	4.7273	0.0362	4.6562	4.7984
Water flow improvement	3.8611	0.0564	3.7502	3.9720
Recreational conditions	3.2879	0.0650	3.1600	3.4157
Vegetation restoration	3.8056	0.0614	3.6848	3.9263
Wildlife habitat improvement	3.2323	0.0679	3.0988	3.3658
Increasing fish in the river	3.1919	0.0687	3.0567	3.3270
Ecological condition and food web	4.0076	0.0575	3.8945	4.1206
Improve irrigation conditions	4.1237	0.0552	4.0152	4.2322
Hydro-electricity improvement	3.3384	0.0762	3.1885	3.4882
Restoration fee	2.9646	0.0703	2.8264	3.1029

**Table 6:** Results obtained using the random parameter logit model.

Attributes	Coefficient	Standard Error	*p*-Value
Mean (standard error) for non-random parameters			
Payment	–0.0261 ***	0.0025	0.000
ASC	0.2164 *	0.1197	0.071
Mean (standard error) for random parameters			
Forest cover ratio	0.2574 ***	0.0510	0.000
Water quality	–2.3980 ***	0.3260	0.000
Water quantity (proportion of the national average)	0.3074 ***	0.0589	0.000
Erosion area	0.1177 ***	0.0199	0.000
Erosion intensity	0.6150 ***	0.0948	0.000
Natural landscape	0.0985 ***	0.0132	0.000
Condition for eco-tourism and parks	0.0849 ***	0.0194	0.000
Standard deviations for random parameters			
Forest cover ratio	0.6265 ***	0.0764	0.000
Water quality	3.5296 ***	0.3234	0.000
Water quantity (proportion of the national average)	0.6830 ***	0.0754	0.000
Erosion area	0.2008 ***	0.0364	0.000
Erosion intensity	0.9796 ***	0.1794	0.000
Natural landscape	0.0907 ***	0.0183	0.000
Condition for eco-tourism and parks	0.2395 ***	0.0349	0.000
Model statistics			
Log-likelihood	–2194.4834		
LR chi^2^(7)	844.55		
Prob > chi^2^	0.0000		
Number of observations	900		

**Note**: *** if *p* < 0.01, and * if *p* < 0.1.

**Table 7:** Implicit prices (marginal willingness to pay (WTP)) for the ecological attributes.

Ecological Attribute	Implicit Price	95% Confidence Interval	
Forest cover ratio	9.87	6.04	13.71
Water quality	–91.99	–116.50	–67.49
Water quantity (proportion of the national average)	11.79	7.37	16.22
Erosion area	4.52	3.02	6.01
Erosion intensity	–23.59	–30.72	–16.47
Natural landscape	3.78	2.79	4.77
Condition for eco-tourism and parks	3.26	1.80	4.72
